# Pulmonary embolism in non-brain tumor patients after surgery—a retrospective study in China

**DOI:** 10.1186/s12957-016-1074-3

**Published:** 2017-01-14

**Authors:** Ren-Xiong Chen, Hong-Zhi Wang, Jun Dong, Hong Ren, Xiao-Jie Chen, Jia-Xuan Xu, Yong Yang, Guo-Dong Wang

**Affiliations:** Critical Care Medicine, Key Laboratory of Carcinogenesis and Translational Research (Ministry of Education), Peking University Cancer Hospital and Institute, Beijing, 100142 China

**Keywords:** Pulmonary embolism, Tumor patients, Surgery

## Abstract

**Background:**

The incidence rate of pulmonary emboli (PE) is high in tumor patients; however, the morbidity and mortality associated with the development of PE after tumor surgery are unknown. We studied the clinical profiles and outcomes of patients with PE after non-brain tumor surgery.

**Methods:**

We retrospectively screened 55,967 patients who underwent non-brain tumor surgery at the Peking University Cancer Hospital from January 2008 to June 2015. Among them, 76 patients who were diagnosed with PE were enrolled in our study. Factors impacting the overall survival at 90 days were analyzed. A Kaplan-Meier curve was plotted for time to death or until day 90. Cox proportional hazard modeling was performed for univariate- and multivariate-adjusted factor analyses.

**Results:**

The morbidity rate was approximately 135.8 per 100,000 non-brain tumor surgery patients (possibly underestimated). When treated, seven patients had major bleeding, and 14 patients had clinically relevant non-major bleeding, which represented 9.2 and 18.4% of all the patients, respectively. The 3-month overall mortality rate was 11.8% in our study. The Acute Physiology and Chronic Health Evaluation II (APACHE II) score and platelet distribution width (PDW) were independent risk factors for the prognosis of PE after non-brain surgery (*P* values of 0.001 and 0.016, respectively).

**Conclusions:**

Treatment of PE in non-brain tumor surgical patients remained a challenge due to the high bleeding rate. The APACHE II score and PDW were independent prognostic factors of survival in patients with PE after non-brain tumor surgery; however, the study power was limited.

## Background

Venous thromboembolism (VTE) includes deep vein thrombosis (DVT) and pulmonary embolism (PE). Pulmonary emboli account for an increasing proportion of venous thromboemboli with increasing patient age. Venous thromboembolism is the third most common cardiovascular disease [1, 2]. There are approximately 300,000–600,000 cases per year in the USA, resulting in 60,000–100,000 deaths each year [3]. Pulmonary embolism-related deaths in the USA may exceed myocardial infarction-related and stroke-related deaths [4]. Although less common in certain regions, such as Asia, venous thromboembolism is a worldwide problem, particularly in people with known risk factors [5, 6]. In China, the overall annual incidence of PE is 3.9 per 100,000 individuals and the hospital mortality rate associated with PE is 23.8% [5].

Surgery and malignancy increase the risk of VTE [7, 8]. The risk of VTE increases over 90-fold during the first 6 weeks after cancer surgery, compared with that observed in healthy controls, and is second only to the risk of VTE after hip or knee replacement surgery [9]. The risk of venous thrombosis in cancer patients depends on the tumor type and stage, treatment measures, and patient-associated factors [10]. The mechanisms may involve mucin production by tumors, exposure of tissue factor-rich surfaces and tissue factor-bearing microparticles, cysteine proteinase production leading to thrombin generation, and local hypoxia [11, 12].

However, previous studies have not analyzed patients according to their status, e.g., medical or surgery patients. Because of the high risk of bleeding, therapy for patients with PE after surgery is different from medical patients. The aim of this study was to explore the clinical profiles and outcomes of patients with PE after non-brain tumor surgery.

## Methods

### Study population

From January 2008 to June 2015, 55,967 patients who underwent surgery for the treatment of tumors at the Peking University Cancer Hospital were retrospectively screened. The diagnosis of PE was made according to the American College of Chest Physicians, ninth edition (ACCP 9th Ed.) guidelines. A total of 76 cases were included for analysis in our study.

### Definitions

Patients who underwent fibrinolysis received a single weight-based intravenous administration (over a period of 2 h) of the fibrinolytic agent urokinase.

Those patients were administered nadroparin calcium first. We defined adequate anticoagulation treatment as the dose of nadroparin calcium for 0.08–0.1 mL/10 kg, every 12h (q12h), and reduced anticoagulation treatment as less than 0.08 mL/10 kg, q12h.

Bleeding was considered major if it was clinically overt and associated with a decrease of 2 g per deciliter (or more) according to the hemoglobin level, led to the transfusion of 2 or more units of red blood cells, was retroperitoneal or intracranial, occurred in a critical organ, or contributed to death. Bleeding episodes that were clinically relevant but did not qualify as major (e.g., epistaxis that required intervention, formation of a large hematoma visible on the skin, or spontaneous macroscopic hematuria) were classified as clinically relevant non-major bleeding.

### Statistical analyses

Values are expressed as the means (ranges) unless otherwise stated. The effect of factors influencing the overall survival at 90 days was analyzed. A Kaplan-Meier curve was plotted for time to death or until day 90. Cox proportional hazard modeling was performed for univariate- and multivariate-adjusted factor analyses. The forward stepwise method was applied during the multivariate analysis. Statistical analyses were performed using SPSS version 18.0. *P* values less than 0.05 (two-tailed) were considered significant.

## Results

### Patient characteristics

Seventy-two patients were diagnosed with PE by computed tomographic pulmonary angiography (CTPA). Two patients were diagnosed via positive compression venous ultrasonography with decreased peripheral blood oxygen saturation because CTPA was unsafe for these patients. Two patients died quickly in the general ward (making an examination impossible). In these patients, PE was diagnosed via clinicians with expert clinical experience. The main baseline characteristics of the included patients are presented in Table 1. The clinical presentations and diagnoses of the included patients are listed in Table 2.

**Table 1:** Patients with PE

Items	Results
Age, years (mean ± SD)	64.1 ± 10.1
Male/female (number)	34/42
BMI, kg/m^2^ (mean ± SD)	26.0 ± 3.5
Tumor (number)	
Breast cancer	20
Digestive system cancer^a^	33
Thoracic tumor^b^	18
Genital urinary tract tumor^c^	4
Melanoma	1
Coexisting conditions (number)	
Hypertension	34
Diabetes	13
Varicose vein of lower limb	10
Accompanying infection (number)	
Pulmonary infection	5
Abdominal infection	9
Pleural infection	2
Surgery incision infection	1
ICU admission (number)	56
Hemoglobin before surgery, g/L (mean ± SD)	131.4 ± 18.1

*PE* pulmonary embolism, *BMI* body mass index, *SD* standard deviation

^a^Including 4 cases of esophageal cancer, 12 cases of gastric cancer, 10 cases of colorectal cancer, 4 cases of liver cancer, 2 cases of pancreatic cancer, and 1 case of primary peritoneal carcinoma

^b^Including 13 cases of lung cancer, 4 cases of benign lung tumors, and 1 case of thymoma

^c^Including two cases of endometrial carcinoma, one case of ovarian cancer, and one case of uterine cancer

**Table 2:** Diagnosis of PE

Diagnosis	Number of patients
Symptoms^a^	
Dyspnea	50
Syncope	7
Palpitation	5
Lower leg pain	6
Cardiac arrest	5
Chest pain	2
Asymptomatic	15
Electrocardiogram^b^	
Sinus tachycardia	22
S1Q3T3	5
ST-T changes	10
Low voltage	5
Atrial fibrillation	3
Normal	21
Gas analysis^c^	
PCO_2_ < 35 mmHg	12
PO_2_/FiO_2_ ≤ 300 mmHg	57
Lac ≥ 2 mmol/L	19
D-Dimer elevated	63
Pulmonary artery hypertension^d^	3
Positive troponin^e^	2
Compression venous ultrasonography^f^	
Lower extremity venous thrombosis	32
Internal jugular and subclavian vein thrombosis	3
APACHE II score > 15	13

*APACHE II score* Acute Physiology and Chronic Health Evaluation II score

^a^Some patients had more than one symptom

^b^Data were available for 59 patients

^c^Data were available for 68 patients

^d^Data were available for 24 patients

^e^Data were available for 60 patients

^f^Data were available for 70 patients

### Treatment, bleeding, and recurrence

All the patients were provided mechanical prophylaxis after surgery. However, only 16 patients received prophylactic anticoagulation drugs before the diagnosis of PE. Every patient was provided oxygen after the diagnosis of PE. Among them, six cases underwent tracheal intubation ventilator-assisted breathing; four cases used non-invasive ventilator-assisted breathing initially (and two cases improved); the other two patients finally advanced to tracheal intubation ventilator-assisted breathing.

In this group, two patients did not receive any anticoagulant or thrombolytic agents, and two patients were administered urokinase (20,000 U/kg of body weight) for intravenous thrombolysis within 2 h. Sequentially, the patients were administered nadroparin calcium. Among the patients who used anticoagulants as an initial therapy, one patient received fondaparinux because of heparin-induced thrombocytopenia (HIT) after the prevention of anticoagulant therapy with nadroparin calcium, and the others were initially administered nadroparin calcium. Because of the high risk of bleeding after surgery, more than half of the patients in this group received a reduced dosage of anticoagulant therapy.

Thereafter, 33 patients were transitioned to warfarin by mouth or nutrient line when permitted. The international normalized ratio (INR) was monitored. When the INR reached 2.0 or higher for at least 24 h, unfractionated heparin or nadroparin calcium was discontinued. Forty patients continued to use nadroparin calcium as anticoagulation therapy, and one patient used fondaparinux.

The frequency of bleeding is listed in Table 3. During the course of treatment, seven patients experienced major bleeding. Therefore, two patients stopped anticoagulation therapy and five patients decreased the dosage of anticoagulation therapy and were administered a blood transfusion. A total of 14 patients experienced clinically relevant non-major bleeding and were adjusted to a reduced dosage of anticoagulation therapy. Ultimately, all patients stopped bleeding.

**Table 3:** Treatment and bleeding

Initial therapy (numbers)		Major bleeding	Clinically relevant non-major bleeding
Thrombolysis (2)		1	0
Adequate anticoagulation (31)		1	5
Reduced anticoagulation (41)		5	9

Five cases experienced a recurrence of VTE within 3 months. Among them, two cases were administered the intravenous thrombolytic agent urokinase as a remedial treatment and three cases received unfractionated heparin. At the same time, one patient received an inferior vena cava filter after the recurrence of VTE.

### Survival analysis

**Fig. 1:**
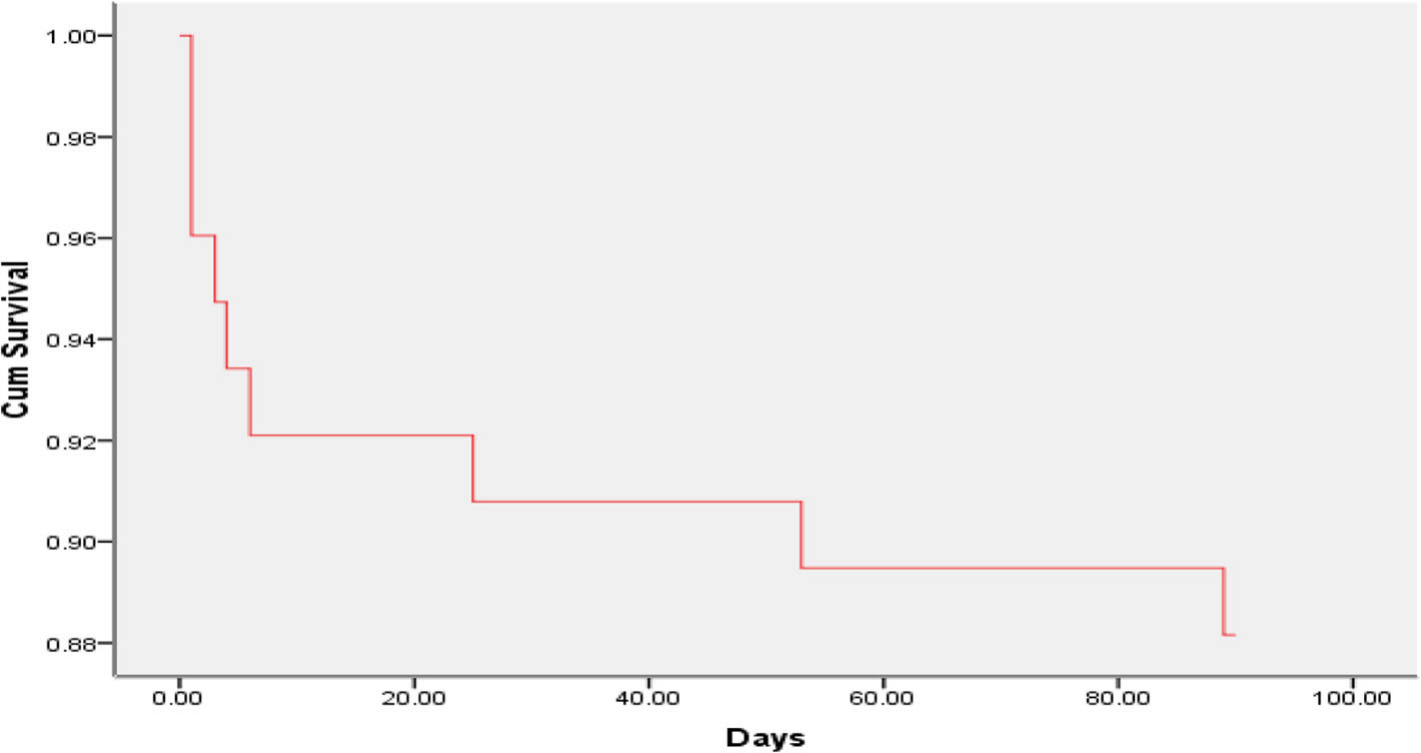
Kaplan-Meier survival curve of overall survival at 90 days

The patients were followed up for 3 months, and no patients were lost to follow-up. The 3-month mortality rate was 11.8% (9/76). Survival curves are shown in Fig. 1. Age, body mass index (BMI), Acute Physiology and Chronic Health Evaluation II (APACHE II) score, platelet distribution width (PDW), and mean platelet volume divided by platelet (MPV/PLT) were analyzed in the multiple Cox regression analysis. The APACHE II score and PDW were independent risk factors for the prognosis of PE after surgery, and the relative risk values were 1.229 and 1.840, respectively (Table 4). The area under the curve (AUC) of the APACHE II scores according to the Receiver Operating Characteristic (ROC) curve was 0.923. If the cutoff APACHE II score was 12, the sensitivity was 1 and the specificity was 0.716. The AUC of PDW according to the ROC curve was 0.706. If the cutoff PDW was 12.4, the sensitivity was 0.778 and the specificity was 0.642.

**Table 4:** Cox regression analysis

	RR	95% CI	*P* value
APACHE II score	1.229	1.127–1.341	0.001
PDW	1.840	1.118–3.027	0.016

## Discussion

The morbidity rate of PE was approximately 135.8 per 100,000 patients in our study. In this study, digestive system cancer (43.4%) accounted for the largest proportion, followed by breast cancer (26.3%). These tumors are the most common tumors in our hospital. There were no patients with PE after orthopedic surgery in our hospital during the study years, which might be attributed to positive preventive anticoagulation. The clinical signs and symptoms of PE were non-specific. PE should be suspected based on dyspnea, chest pain, pre-syncope, syncope, and/or hemoptysis. Arterial hypotension and shock were rare but important clinical presentations because they indicated a severely reduced hemodynamic reserve [13]. In our study, dyspnea was the most common presenting symptom, observed in 65.8% of the patients, followed by a lack of symptoms with decreasing oxygen saturation.

In our study, 72 patients were diagnosed by computed tomographic pulmonary angiography; the other 4 patients could not be safely transported for CTPA. Computed tomographic pulmonary angiography seems to be the most useful method for the diagnosis of PE in critically ill patients. For patients who are so critically ill that transport is unsafe or unfeasible, thrombolytic therapy should be considered if there are clear signs of right ventricular overload according to bedside echocardiography [14]. A positive result on a lower extremity Doppler ultrasound can also be a great help in the decision for treatment.

The choice of treatment for PE depends on the estimated risk of poor outcome. The presence of hypotension is the most significant predictor of poor outcome and is common in patients with massive PE. Currently, in patients with acute PE associated with hypotension who do not have a high risk of bleeding, systemically administered thrombolytic therapy is proposed [15]. For patients with a contraindication to anticoagulation and thrombolytic therapy, surgical embolectomy and catheter-based therapies are options. However, the role of inferior vena cava filters, catheter-based interventions, and surgical embolectomy in life-threatening PE has not yet been absolutely defined. Muriel et al. reported that in patients presenting with VTE and a significant bleeding risk, inferior vena cava filter insertion was associated with a lower risk of PE-related death and a higher risk of recurrent VTE (compared with anticoagulant therapy) [16].

Normotensive patients with evidence of right ventricular (RV) dysfunction as assessed by echocardiography comprise the sub-massive category and have an intermediate risk of poor outcomes. Clinically, patients with sub-massive PE are difficult to distinguish from patients with low-risk PE. Some studies reported that cardiac troponin and brain natriuretic peptide could raise the suspicion that a patient had sub-massive PE [17–19]. The role of fibrinolytic therapy in patients with intermediate-risk PE is controversial. According to one study [20], in patients with intermediate-risk PE, fibrinolytic therapy could reduce early death but increases the risk of major bleeding and stroke. During recent years, echocardiography and brain natriuretic peptide (BNP) have not been examined routinely in our hospital. Many data were missing, and we could not stratify non-high-risk PE patients into intermediate-risk and low-risk PE patients. However, this did not influence our treatment of those patients. All patients initially received anticoagulation therapy.

Anticoagulation is the foundation therapy for PE. Low-molecular-weight heparins and fondaparinux were preferred over unfractionated heparin due to their usability [21–23]. All patients with PE in our study had a high risk of bleeding after surgery. We should consider the balance of bleeding risk versus benefit for each patient. However, there was no compelling evidence to validate a scoring system to predict the bleeding risk for patients with VTE. Seven patients experienced major bleeding, and 14 patients experienced clinically relevant non-major bleeding, which accounted for 9.2 and 18.4% of all patients, respectively. This rate is higher than other reports. As stated above, all patients stopped bleeding via conservative therapy and no bleeding-related deaths occurred.

The 3-month overall mortality rate was previously reported to be approximately 15 to 18% [24]; the values found in our study were lower. Shock upon presentation was associated with an increase in mortality by a factor of 3–7 [24]. Naess et al. reported that secondary cancer VTE is associated with increased mortality relative to secondary non-cancer VTE and idiopathic VTE [25]. According to a study by Yardan et al. [26], a high MPV/PLT ratio is associated with RV dysfunction and clinical severity in patients with acute PE and a low MPV/PLT level might be an indicator of low bleeding risk in patients with acute PE. In our study, the APACHE II score and PDW were independent prognostic factors of survival (but not MPV/PLT). The total number of patients in our study was limited, and further research is needed.

The risk of recurrence was particularly high among patients with cancer [14]. The probability of recurrent thromboembolism at 6 months was 17% in the oral-anticoagulant group and 9% in the low-molecular-weight heparin group [27]. The recurrence rate of VTE was approximately 6.5% within 90 days in our study. Two patients died after VTE recurrence. In the recurrent patients, two cases received intravenous unfractionated heparin for the treatment of PE and they ultimately survived. This therapy provides another option for patients with recurrence; however, more studies are needed to support this treatment course.

Prophylaxis remained underutilized in patients who were admitted to the hospital with a moderate or high risk of venous thromboembolism and was probably due to the concern regarding the risk of bleeding. We should propose guidelines for the prophylaxis and management of PE after surgery. More active education is required to raise awareness and to ensure the implementation of these guidelines, which may reduce the burden of PE in patients with tumors after surgery. For patients with a high risk of VTE who are undergoing abdominal or pelvic surgery for cancer, the ACCP 9th Ed. guidelines recommend extended-duration, postoperative, pharmacologic prophylaxis (4 weeks) with LMWH (in contrast to limited-duration prophylaxis) [15].

Currently, the USA has approved one direct thrombin inhibitor (dabigatran) and three factor Xa inhibitors (apixaban, rivaroxaban, and edoxaban) for the treatment of acute VTE. In a series of important studies, each of these agents has been demonstrated to be at least as safe and effective for the treatment of acute VTE as the traditional bridging strategy (parenteral anticoagulation/warfarin) [28–31]. These drugs do not require any coagulation monitoring in the laboratory and may be accepted by most people in the future.

Several limitations should be mentioned. First, this study was a retrospective study. A prospective study with a control group consisting of patients with PE without tumors and without operations may be more useful. Second, many data were missing, especially data regarding ultrasound cardiogram (UCG). Third, the prevalence of PE may have been underestimated because no autopsies were performed and not all patients underwent CTPA. Finally, the sample size in our COX analysis was small, increasing the risk of a type 2 error due to a lack of statistical power. Larger studies are needed to address the challenging issues during the treatment of tumor surgical patients with PE.

## Conclusions

The treatment of PE in surgical patients with tumors remains a clinical challenge due to the high bleeding rate. The APACHE II score and PDW were independent prognostic factors of survival in patients with PE after non-brain surgery; however, the statistical power was limited.

## Abbreviations

ACCP 9th Ed.: American College of Chest Physicians, ninth edition
APACHE II score: Acute Physiology and Chronic Health Evaluation II score
AUC: Area under the curve
BMI: Body mass index
BNP: Brain natriuretic peptide
CTPA: Computed tomographic pulmonary angiography
DVT: Deep vein thrombosis
MPV/PLT: Mean platelet volume divided by platelet count
PDW: Platelet distribution width
PE: Pulmonary embolism
ROC: Receiver Operating Characteristic curve
UCG: Ultrasound cardiogram
VTE: Venous thromboembolism
